# Diversifying Selection on the Thrombospondin-Related Adhesive Protein (TRAP) Gene of *Plasmodium falciparum* in Thailand

**DOI:** 10.1371/journal.pone.0090522

**Published:** 2014-02-28

**Authors:** Jun Ohashi, Yuji Suzuki, Izumi Naka, Hathairad Hananantachai, Jintana Patarapotikul

**Affiliations:** 1 Molecular and Genetic Epidemiology, Faculty of Medicine, University of Tsukuba, Ibaraki, Japan; 2 Department of Microbiology and Immunology, Faculty of Tropical Medicine, Mahidol University, Bangkok, Thailand; University at Buffalo, United States of America

## Abstract

Sporozoites of *Plasmodium falciparum* are transmitted to human hosts by Anopheles mosquitoes. Thrombospondin-related adhesive protein (TRAP) is expressed in sporozoites and plays a crucial role in sporozoite gliding and invasion of human hepatocytes. A previous study showed that the TRAP gene has been subjected to balancing selection in the Gambian *P. falciparum* population. To further study the molecular evolution of the TRAP gene in *Plasmodium falciparum*, we investigated TRAP polymorphisms in *P. falciparum* isolates from Suan Phueng District in Ratchaburi Province, Thailand. The analysis of the entire TRAP coding sequences in 32 isolates identified a total of 39 single nucleotide polymorphisms (SNPs), which comprised 37 nonsynonymous and two synonymous SNPs. McDonald–Kreitman test showed that the ratio of the number of nonsynonymous to synonymous polymorphic sites within *P. falciparum* was significantly higher than that of the number of nonsynonymous to synonymous fixed sites between *P. falciparum* and *P. reichenowi*. Furthermore, the rate of nonsynonymous substitution was significantly higher than that of synonymous substitution within Thai *P. falciparum*. These results indicate that the TRAP gene has been subject to diversifying selection in the Thai *P. falciparum* population as well as the Gambian *P. falciparum* population. Comparison of our *P. falciparum* isolates with those from another region of Thailand (Tak province, Thailand) revealed that TRAP was highly differentiated between geographically close regions. This rapid diversification seems to reflect strong recent positive selection on TRAP. Our results suggest that the TRAP molecule is a major target of the human immune response to pre-erythrocytic stages of *P. falciparum*.

## Introduction

Malaria infection is initiated during the sporozoite stage of the *Plasmodium* when the parasite is injected into the bloodstream by an infected female Anopheles mosquito. Sporozoites quickly pass into the liver, where they infect hepatocytes. Thrombospondin-related adhesive protein (TRAP) [1] is stored in the micronemes of sporozoites [2] and is released onto the cell surface at the anterior tip on contact of sporozoites with host cells [3]. TRAP has been shown to play a crucial role in sporozoite gliding, motility, and invasion of hepatocytes [4]–[6].

*P. falciparum*-infected hepatocytes can be recognized and eliminated by cytotoxic T lymphocytes (CTLs). The CTL-based adaptive immune response appears to be important for protection against the pre-erythrocytic stages of malaria. Presentation of pathogen-derived peptides by human leukocyte antigen (HLA) class I molecules on infected cells to T cell receptors (TCRs) on CTLs is a central event in CTL-mediated elimination of target cells. TRAP is one of the potential proteins that has epitopes presented by HLA class I molecules on infected hepatocytes to TCRs on CTLs. This has been supported by the identification of a CTL response to certain TRAP epitopes in humans [7]–[11]. These observations raise the question of whether the TRAP gene of *P. falciparum* has been under strong selective pressure due to human immune response.

A previous study [12] revealed a higher ratio of the number of nonsynonymous to synonymous polymorphisms of TRAP within the Gambian *P. falciparum* population compared with that of nonsynonymous to synonymous substitutions between the Gambian *P. falciparum* population and *P. reichenowi*. A significant difference was observed in this ratio (i.e., the former was higher than the latter) by the McDonald–Kreitman test, suggesting that the TRAP gene is under diversifying selection in the Gambian *P. falciparum* population. Furthermore, the observed Tajima’s *D* and Fu and Li’s *F* values for TRAP in the Gambian *P. falciparum* population were significantly higher than those from a coalescent simulation under neutrality, suggesting that the TRAP gene is subject to balancing selection, which maintains diversity in the Gambian *P. falciparum* population [12]. However, no evidence for balancing selection was reported for samples obtained from Tak province, Thailand [12]. Thus, it remains unclear whether positive selection acts on the TRAP gene of *P. falciparum* in other geographic regions apart from Africa. The aims of the present study were to investigate whether the TRAP gene is under diversifying selection in Thai *P. falciparum* and to elucidate how TRAP gene is differentiated among *P. falciparum* populations. Our results would be helpful not only for understanding the molecular evolution of the TRAP gene in *P. falciparum* but also for designing peptide vaccines based on the TRAP antigen.

## Results

### *TRAP* Polymorphisms of *P. falciparum* in Thailand

**Figure 1:**
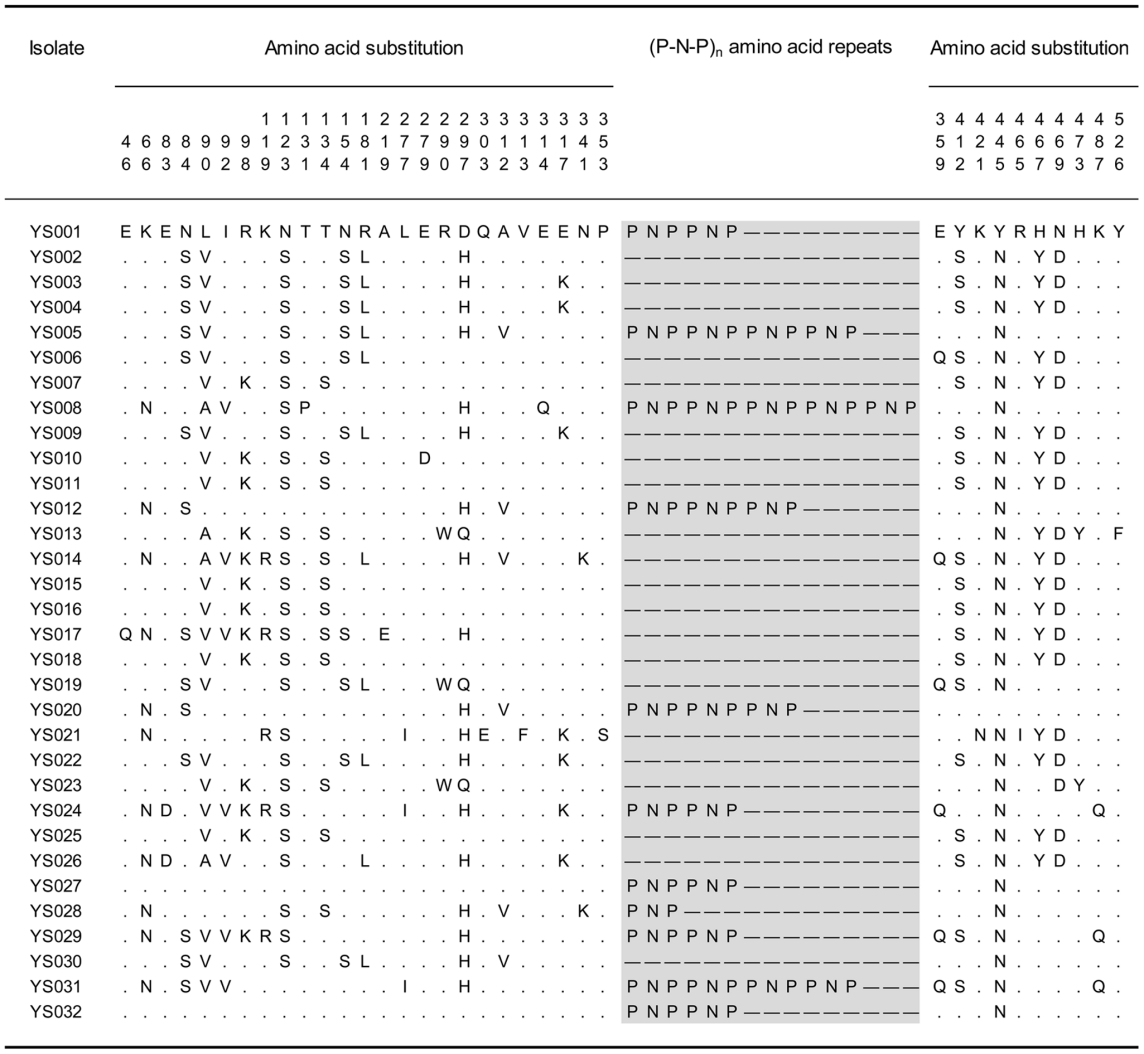
Alignment of amino acid sequences of the TRAP gene of 32 *P. falciparum* isolates. Only polymorphic residues in 32 isolates are shown. Residues identical with those of YS001 are indicated by dots, and deletions in the region of the P-N-P repeat are indicated by dashes.

To detect TRAP polymorphisms within *P. falciparum* isolates from Suan Phueng District in Ratchaburi Province, Thailand, we performed direct PCR sequencing of DNA samples from 32 patients with *P. falciparum* malaria. The sequence reads obtained from direct sequencing of both DNA strands did not show ambiguous electropherogram peaks at any site, indicating that the single or most abundant allele in each blood sample was successfully determined. These 32 single allele sequences of the entire coding region of the TRAP gene (GenBank Accession No. AB807828–AB807859) were statistically analyzed as below, and among them, 23 different allelic haplotypes were identified (Figure 1).

**Figure 2:**
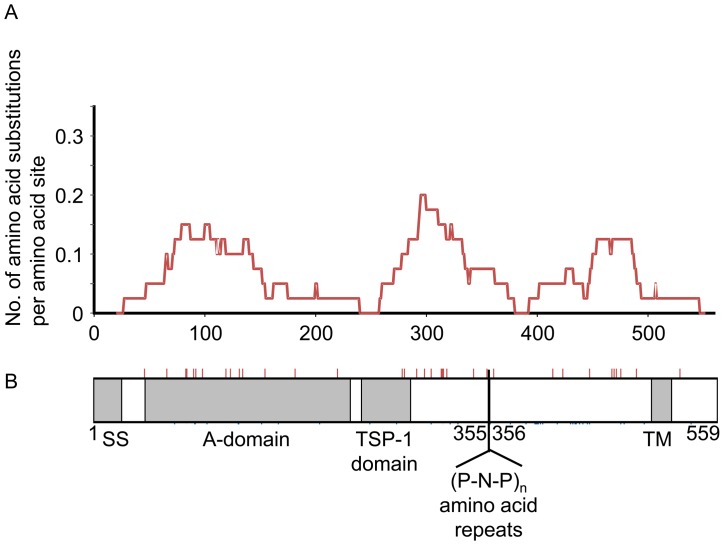
Polymorphic amino acid residues of the TRAP gene in 32 *P. falciparum* isolates. A, Density of polymorphic amino acid residues in the TRAP gene. A sliding window analysis of amino acid polymorphisms (substitutions) was conducted using a sliding window size of 20 amino acids and a step size of one amino acid. B, Schematic representation of the TRAP domain composition, signal sequence (SS), von Willebrand factor A domain (A domain), thrombospondin type 1 domain (TSP-1), and transmembrane domain (TM). The proline-rich repeat region lies between TSP-1 and TM. Positions of polymorphic amino acid residues are indicated by the thin red line above the box.

A total of 39 single nucleotide polymorphisms (SNPs), comprising 37 nonsynonymous and two synonymous SNPs, were found in the coding region of the TRAP gene of 32 Thai *P. falciparum* isolates. Amino acid alterations caused by single point mutations included 33 diallelic and two triallelic amino acid polymorphisms (Figure 1). Therefore, 35 amino acid residues were polymorphic among a total of 559 residues of the TRAP gene (Figures 1 and 2B). The density of polymorphic residues was relatively high in von Willebrand factor A domains and in the proline-rich repeat regions (Figure 2A), with 13 and 17 amino acid polymorphisms in A domains and proline-rich repeat regions, respectively. In contrast, the N-terminal signal sequence and transmembrane domain had no amino acid polymorphisms, apparently reflecting the difference in the functional constraints among domains.

In addition to nonsynonymous SNPs, the TRAP gene had a repeat region with a nonanucleotide repeat unit encoding proline-asparagine-proline (P-N-P; Figure 1). The number of repeats varied from 0 to 5. As found in a previous study of TRAP polymorphisms in *P. falciparum* field isolates from Tak Province, Thailand [13], only simple P-N-P repeats were detected in the present samples from Ratchaburi Province, Thailand. Unlike Thai *P. falciparum*, glutamate-asparagine-proline-serine-asparagine-proline (E-N-P-S-N-P) repeats were observed in the Gambian *P. falciparum* population [12]. The allelic haplotypes containing P-N-P and E-N-P-S-N-P repeats exclusively have P and S, respectively, at position 353 in the Gambian *P. falciparum* population, whereas no haplotypes contain both P-N-P and E-N-P-S-N-P repeats in any *P. falciparum* population. Because the S allele at position 353 is found in the Thai *P. falciparum* population (e.g., YS021 in Figure 1), allelic haplotypes containing E-N-P-S-N-P repeats may also exist at a low frequency in Thailand or may appear in the future. P-N-P repeat regions were excluded from the analyses below unless otherwise stated.

### Polymorphic Sites within Species and Fixed Sites between Species

The McDonald–Kreitman test [14] for the aligned sequence data of TRAP sequences of 32 Thai *P. falciparum* isolates and *P. reichenowi* (GenBank Accession No. AY880881) revealed that the ratio of the number of nonsynonymous (N) to synonymous (S) polymorphic sites within *P. falciparum* (*P*_N_/*P*_S_ = 36/2) was significantly higher than that of the number of nonsynonymous to synonymous fixed sites between Thai *P. falciparum* and *P. reichenowi* (*D*_N_/*D*_S_ = 36/12; *P*-value = 0.018 by Fisher’s exact test).

### Nonsynonymous and Synonymous Substitution Rates within the Thai *P. falciparum* Population

**Figure 3:**
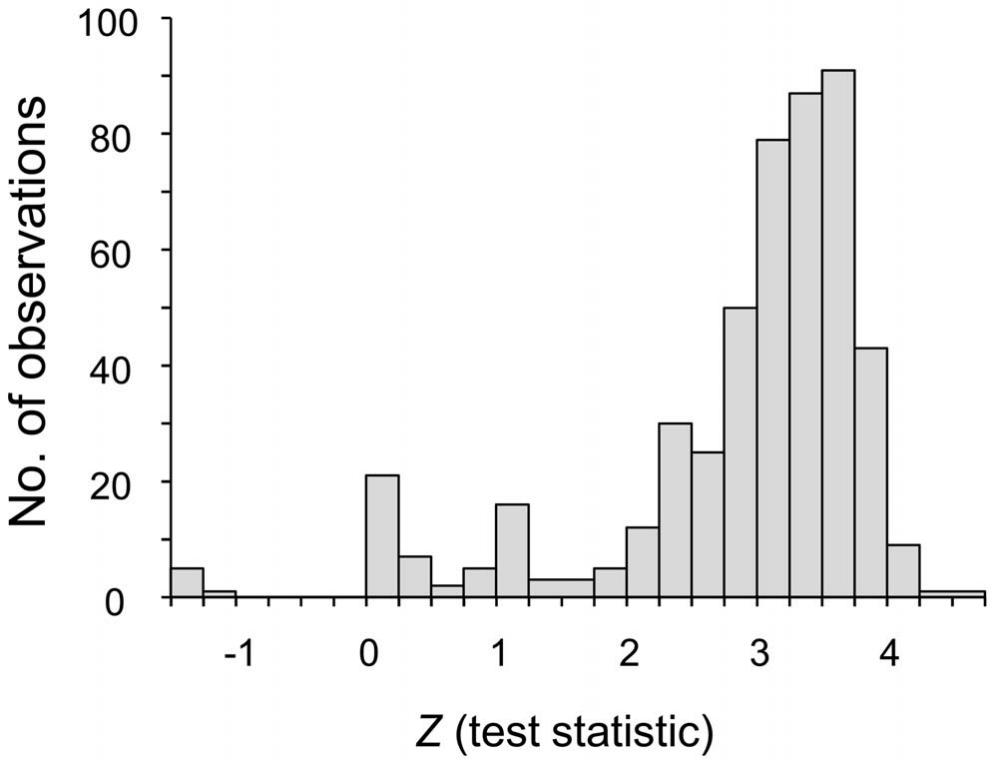
Distribution of the *Z*-test statistic for pairwise comparison of the rate of nonsynonymous substitution (*d*_N_) with that of synonymous substitution (*d*_S_) among 32 *P. falciparum* isolates.

The significantly higher ratio of the number of nonsynonymous to synonymous polymorphisms within *P. falciparum* compared with that of nonsynonymous to synonymous substitutions between *P. falciparum* and *P. reichenowi* (i.e., *P*_N_/*P*_S_>*D*_N_/*D*_S_) in the McDonald–Kreitman test implies that either purifying selection (negative selection) or diversifying selection (positive selection) has been acting on the TRAP gene in *P. falciparum*. To evaluate this, *Z*-test statistic, (*d*_N_ − *d*_S_)/sqrt{var(*d*_N_ − *d*_S_)}, was calculated, where rates of nonsynonymous substitution (*d*_N_) and synonymous substitution (*d*_S_) were calculated for each pair of sequences within Thai *P. falciparum* and the variance of (*d*_N_ − *d*_S_) was computed using the bootstrap resampling method. Most pairs gave positive *Z*-test statistics (Figure 3), and binomial tests revealed that *d*_N_ was significantly higher than *d*_S_ (*P*-value = 8.4×10^−101^). The present results suggest that the TRAP gene in Thai *P. falciparum* has been subject to diversifying selection.

### Neutrality Test based on Frequency Distribution of Segregating Sites

Tajima’s *D* statistic [15] was calculated to test the departure from neutral evolution under constant population size. This test compares population genetic parameters estimated from mean pairwise nucleotide differences with those estimated from the number of segregating sites. Tajima’s *D* value was −0.13 for TRAP sequences of 32 Thai *P. falciparum* isolates. A coalescence-based computer simulation revealed no significant differences in Tajima’s *D* value between observed and expected values under neutrality (*P*-value = 0.86). A sliding window analysis (window size of 250 and step size of 10 bp) of Tajima’s *D* across the entire TRAP gene for 32 Thai *P. falciparum* isolates also showed no remarkable deviation of Tajima’s *D* from 0 (Figure S1). Unlike the Gambian *P. falciparum* population [12], balancing selection, which maintains polymorphisms with intermediate-frequency alleles, may not apparently operate in the Thai *P. falciparum* population.

### Distribution of Allele Frequencies at the P-N-P Repeat

We detected six P-N-P repeat alleles of different lengths (Figure 1). Each P-N-P repeat was encoded by the nonanucleotide sequence motif CCAAATCCA. To determine whether the observed distribution of allele frequencies at the P-N-P repeat deviated from the expected value under neutrality, we performed the Ewens–Watterson homozygosity test [16]. A two-sided test showed neither significant excess nor deficiency of *F* in the observed data (two-sided *P*-value = 0.22), suggesting that the P-N-P repeat is not subject to strong positive selection.

### Genetic Differentiation between *P. falciparum* Populations

**Figure 4:**
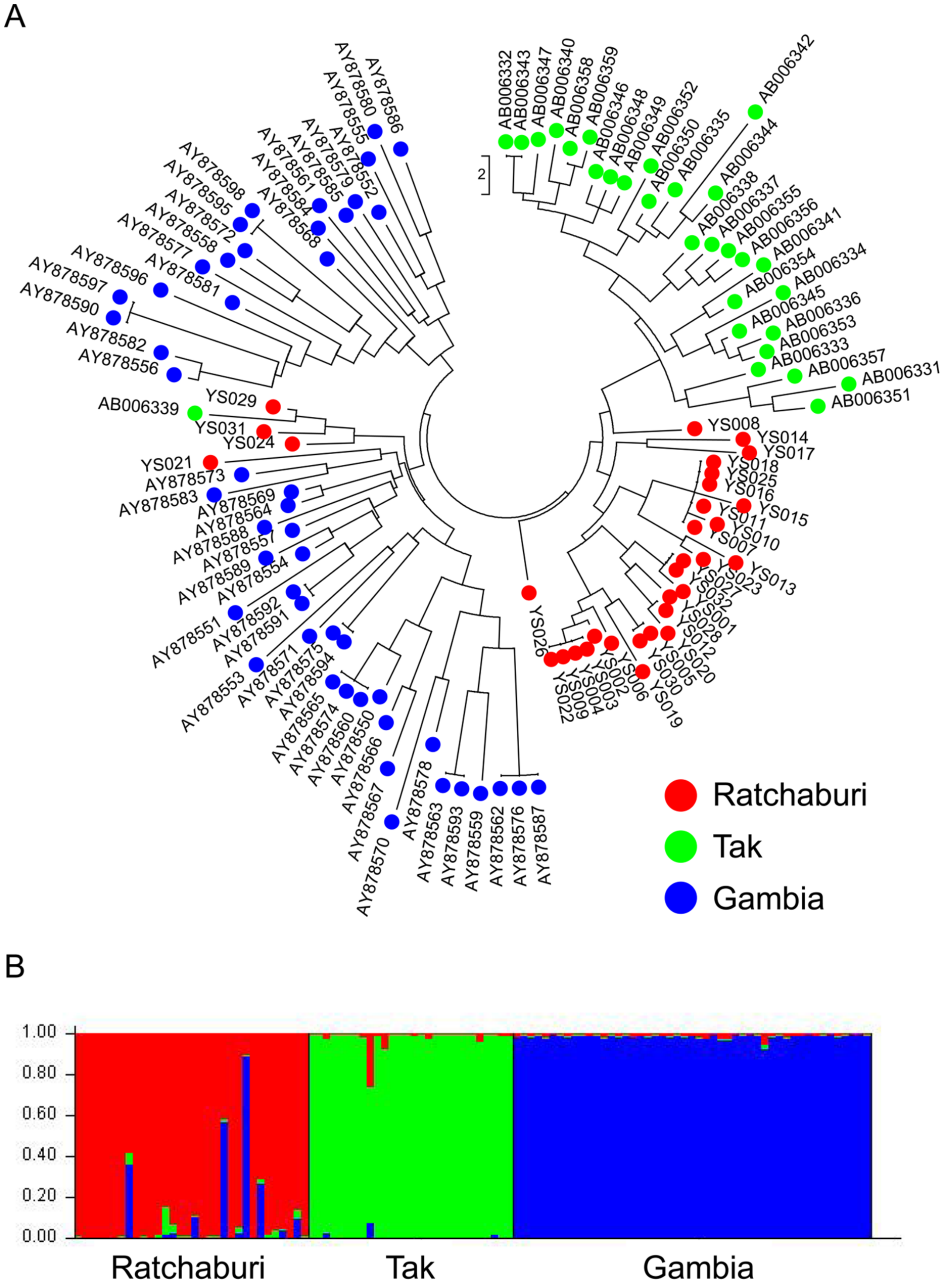
Phylogenetic tree (A) and population structure (B) of the TRAP gene of 110 *P. falciparum* isolates from three geographic regions. Samples included 32 isolates from Ratchaburi Province, Thailand (investigated in this study), 29 from Tak Province, Thailand, and 49 from Gambia. A, The phylogenetic tree of the TRAP sequences was constructed using the neighbor-joining method. B, Membership coefficients from STRUCTURE analysis (*K* = 3) were plotted for 110 isolates. Each isolate is represented by a vertical bar displaying the proportion of membership in each of the three hypothetical clusters.

To elucidate geographic differences in the nucleotide sequence of TRAP between Thailand and Gambia, a phylogenetic tree was constructed using the neighbor-joining method for individual sequences (Figure 4A). A phylogenetic analysis indicated that *P. falciparum* malaria parasites are broadly classified into the following three geographical groups: Ratchaburi, Tak, and Gambia. The STRUCTURE analysis for *K* = 3 also showed three distinct clusters that correspond to the geographic regions where samples were obtained (Figure 4B).

**Figure 5:**
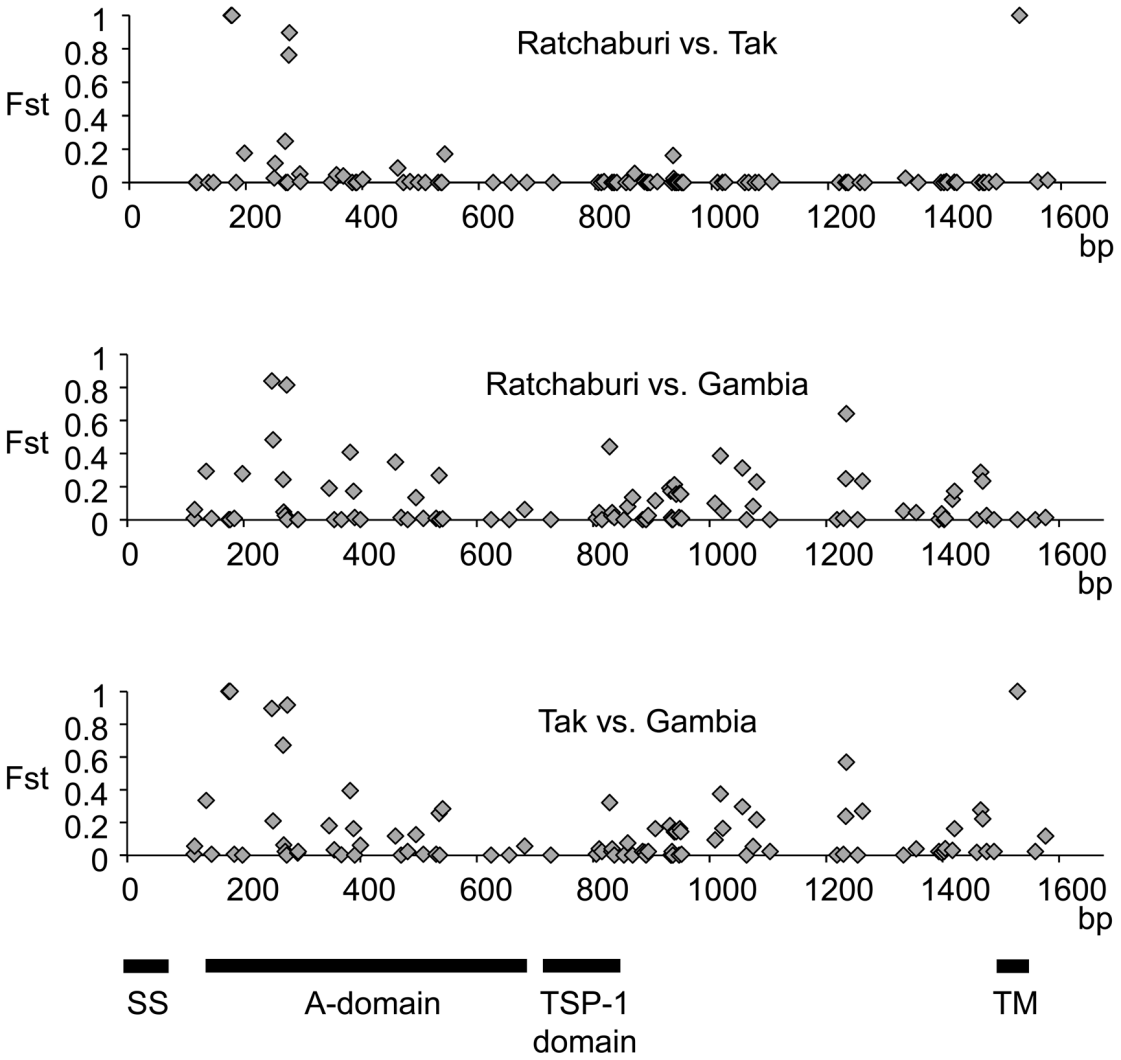
Fst value at each SNP. A total of 94 SNPs were detected in three *P. falciparum* populations. Fst values were calculated for three population pairs (Ratchaburi and Tak, Tak and Gambia, and Gambia and Ratchaburi). The locations of four TRAP domains, signal sequence (SS), von Willebrand factor A domain (A domain), thrombospondin type 1 domain (TSP-1), and transmembrane domain (TM) were indicated by thick horizontal lines.

To examine the genetic differentiation at each SNP, Fst values were calculated for three pairs of *P. falciparum* populations (Figure 5). As expected, *P. falciparum* population in Ratchaburi showed the genetic similarity with that in Tak. Most of SNPs with high Fst values between these Thai *P. falciparum* populations were located in A-domain of TRAP molecule. Although neutrality test using the Tajima’s *D* statistic did not detect signature of positive selection in any region of the TRAP gene (Figure S1), A-domain of TRAP molecule may be evolving rapidly to escape the human immune response.

## Discussion

In the present study, we examined whether the TRAP gene has been subject to positive selection in Thai *P. falciparum*. The ratio of the number of nonsynonymous to synonymous polymorphic sites within *P. falciparum* was significantly higher than that of nonsynonymous to synonymous fixed sites between *P. falciparum* and *P. reichenowi*. This observation excludes the possibility of directional selection after divergence of *P. falciparum* from *P. reichenowi*. The rate of nonsynonymous substitutions was found to be significantly higher than that of synonymous substitutions within Thai *P. falciparum*. Thus, we conclude that diversifying selection has acted on the TRAP gene of *P. falciparum* in Southeast Asia as previously reported in Africa [12].

If diversifying selection has acted on the TRAP gene for a long evolutionary time, the rate of nonsynonymous substitution (*d*_N_) should be higher than that of synonymous substitution (*d*_S_) between *P. falciparum* and *P. reichenowi*, because fixation eventually occurs even under diversifying selection. However, the *d*_N_/*d*_S_ ratio between a Thai *P. falciparum* isolate and *P. reichenowi* was not significantly higher than 1 (data not shown), suggesting that diversifying selection on the TRAP gene may have started to act recently in the Thai *P. falciparum* population.

Of 94 SNPs across the three *P. falciparum* populations, five, eight, and 40 population-specific SNPs were found in Ratchaburi, Tak, and Gambia, respectively (data not shown). In addition, four SNPs were completely differentiated, with only a single allele observed in each population, leading to three distinct clusters corresponding to three geographic regions in both cluster analyses (Figures 4 and 5). This high level of genetic differentiation of TRAP implies the following four possibilities: the first is that the TRAP gene has diversified in a short period of time due to “strong” diversifying selection. The second is that long-term isolation due to the lack of migration of *P. falciparum* along with humans has caused the genetic differentiation. However, with only 300 km between provinces of Ratchaburi and Tak, it seems unlikely that migration has not occurred between *P. falciparum* populations in Ratchaburi province and in Tak province for a long time. The third is that high mutation rate has accelerated the genetic differentiation. However, the mutation rate at TRAP seems not high, since only 48 fixed sites were observed between *P. reichenowi* and *P. falciparum* (*D*_N_ and *D*_s_ in McDonald–Kreitman test). These two parasites are suggested to have diverged at the time of the divergence between humans and chimpanzees [17]. The forth is that the samples in three regions were collected at different times. As shown in a phylogenetic tree (Figure 4A), the genetic differentiation does not come from random genetic drift but from mutation. Considering the low mutation rate at TRAP, the difference in time of sampling would not have largely affected the degree of genetic differentiation. Thus, the high level of genetic differentiation is considered to be one of evidences for strong diversifying selection that has acted on the TRAP gene. To examine how TRAP is markedly differentiated, we would need to compare the degree of differentiation in TRAP with that in other genes.

At the liver stage of malaria infection by *P. falciparum*, *P. falciparum*-derived antigens, including TRAP, are processed through the endogenous pathway, and the resulting antigenic peptide fragments are presented by HLA class I molecules to TCRs on CTLs. Previous studies have indicated that a wide range of pre-erythrocytic TRAP antigens are targeted by naturally acquired CTLs [7]–[11]. Together with the fact that TRAP has CTL epitopes, we conclude that the TRAP molecule is a major target of the human immune response to pre-erythrocytic stages of malaria due to *P. falciparum*.

Amino acid changes in a CTL epitope can affect processing. Previously detected HLA-A2.1-restricted CTL epitopes [7], HLGNVKYLV (residues 3–11) and GIAGGLALL (residues 500–508), and HLA-B8-restricted CTL epitopes [7], [8], ASKNKEKAL (residues 107–115) and KNKEKALII (residues 109–117), were not found to be variable in *P. falciparum* isolates in Ratchaburi province, Thailand as well as Tak province [13], suggesting that malaria vaccines based on these TRAP epitopes would be effective in Thailand. To develop optimal malaria vaccine, more CTL epitopes restricted by various HLA molecules need to be detected in the conserved region of TRAP. Our findings on TRAP polymorphisms will be useful not only for investigating the molecular evolution but also for developing malaria vaccine.

## Materials and Methods

### Ethics Statement

This study was approved by the Institutional Review Board of the Faculty of Tropical Medicine, Mahidol University, and the Research Ethics Committee of the Faculty of Medicine, University of Tsukuba. Written informed consent was obtained from all patients.

### Subjects

A total of 32 adult patients with malaria infection due to *P. falciparum* living in Suan Pung, Ratchaburi, Thailand, participated in this study. All patients underwent treatment at the Hospital for Tropical Diseases, Faculty of Tropical Medicine, Mahidol University, Thailand. Malaria infection by *P. falciparum* was confirmed by positive blood smear results for the asexual form of *P. falciparum*.

### Sequencing Analysis of the Entire *TRAP* Gene from Genomic DNA Samples

Genomic DNA was extracted from pretreatment peripheral blood samples from patients with malaria due to *P. falciparum* using a QIAamp Blood Kit (Qiagen, Hilden, Germany). Direct PCR sequencing was performed to detect TRAP polymorphisms in Thai *P. falciparum* populations using the following four sets of primers: TRAP1, 5′-GTTGTTGTGTATTTCACTATAT-3′ (forward) and 5′-GAAACAAAGTGACCCCCAAA-3′ (reverse); TRAP2, 5′-TTCAGGATGTTTTGGAGTATCG-3′ (forward) and 5′-GTTCCACAAGAACCCGAAGA-3′ (reverse); TRAP3, 5′-GGATTTGGTGGATTTTCTGG-3′ (forward) and 5′-GAACAAACTTAAGTGATGCACTGTT-3′ (reverse); and TRAP4, 5′-GATCATTTAATTTTCTTGATTCT-3′ (forward) and 5′-TGTGCATGCGTACAAGAAAA-3′ (reverse). These primers were designed based on the reference sequence (GenBank Accession No. U67764.1) and included the entire coding sequence of the TRAP gene of *P. falciparum*. PCR amplification was performed using a GeneAmp PCR System 9700 (Applied Biosystems, Foster City, CA, USA). PCR amplification using TRAP1 or TRAP3 primer pair was performed in a 10 µL reaction mixture containing 0.2 µL (0.2 µM) each of forward and reverse primers, 5 µL 2×AmpliTaq Gold 360 Master Mix, 0.5 µL (5 ng) of genomic DNA, and 4.1 µL dH2O. PCR amplification using TRAP2 or TRAP4 primer pair was performed in a 10 µL reaction mixture containing 0.2 µL (0.2 µM) each of forward and reverse primers, 0.08 µL (2 units) of Fast start Taq DNA polymerase, 0.2 µL (200 µM) of dNTP, 1 µL of 10×PCR buffer with 20 mM MgCl_2,_ 0.5 µL (5 ng) of genomic DNA, and 7.82 µL dH2O. The PCR cycling condition for TRAP1 or TRAP3 primer pair was: 10 minutes initial denaturation at 95°C, followed by 40 cycles with 30 seconds denaturation at 95°C, 30 seconds annealing at 56°C, 45 seconds extension at 72°C and a final 7 minutes extension at 72°C. The PCR cycling condition for TRAP2 or TRAP4 primer pair was: 4 minutes initial denaturation at 95°C, followed by 40 cycles with 30 seconds denaturation at 95°C, 30 seconds annealing at 56°C, 45 seconds extension at 72°C and a final 7 minutes extension at 72°C. The PCR products were sequenced using an ABI Prism 3100 Genetic Analyzer (Applied Biosystems, Foster City, CA, USA). All isolates gave a clear single allele sequence (no superimposed electropherogram peaks at identified polymorphic sites), corresponding to the single or most abundant allele in each blood sample.

### Statistical Analysis

To calculate the proportion of amino acid polymorphisms (substitutions) to amino acid sites (i.e., density of polymorphic amino acid residues), a sliding window analysis of amino acid polymorphisms (substitutions) was conducted using a sliding window size of 20 amino acids and a step size of one amino acid.

The TRAP sequence of *P. reichenowi*, which is a malaria strain found in primates such as chimpanzees and gorillas and is the closest known relative of *P. falciparum*, was retrieved from the GenBank database (GenBank Accession No. AY880881) and then aligned with 32 TRAP sequences of Thai *P. falciparum*. Based on the aligned sequence data, the numbers of nonsynonymous (N) and synonymous (S) polymorphic sites within *P. falciparum* and the numbers of nonsynonymous and synonymous fixed sites between Thai *P. falciparum* and *P. reichenowi* were counted. To test the neutral hypothesis that nonsynonymous and synonymous nucleotide differences occur in the same ratio for fixed (between species) and polymorphic (within species) differences, the McDonald–Kreitman test [14] was performed using DnaSP v5 software [18]. In the present study, two-sided *P*-values were calculated for all statistical tests, and the significance level was set at 0.05.

For each pair of 32 TRAP sequences of Thai *P. falciparum*, the number of nonsynonymous substitutions per nonsynonymous site (*d*_N_) and the number of synonymous substitutions per synonymous site (*d*_S_) were estimated using the Nei–Gojobori method with the Jukes–Cantor model [19]. The variance of (*d*_N_ − *d*_S_) was estimated using the bootstrap resampling method (1,000 resamplings), and *Z*-test statistics were calculated for each pair using MEGA 5.0 software [20]. Binomial tests were used to test the neutral hypothesis that the number of pairs with *d*_N_ higher than *d*_S_ is not different from the number of pairs with *d*_S_ higher than *d*_N_.

Tajima’s *D* test [15] was performed for 32 TRAP nucleotide sequences of Thai *P. falciparum* using Arlequin version 3.5 software [21], where the probability distribution of Tajima’s *D* under neutrality was generated by coalescent simulation. A sliding window analysis of Tajima’s *D* statistic across the TRAP gene for the nucleotide sequences was also performed (a window size of 250 bp and a step size of 10 bp) using a web tool, SLIDER (http://genapps.uchicago.edu/slider/index.html).

Using Arlequin version 3.5 software [22], the Ewens–Watterson test [16], which is based on Ewens sampling theory of neutral alleles [23], was performed to assess whether the observed distribution of allele frequencies at the P-N-P repeat was different from the expected value that was based on neutrality.

After eliminating P-N-P and E-N-P-S-N-P repeats from the TRAP sequences of 110 *P. falciparum* isolates comprising 32 isolates from Ratchaburi Province, Thailand (investigated in this study); 29 from Tak Province, Thailand; and 49 from Gambia, West Africa, the number of pairwise nucleotide substitutions per site was estimated using the two-parameter method of Kimura [24]. The calculation was performed using MEGA 5.0 software [20] with the “Complete Deletion” option. Based on the estimated number of substitutions, a phylogenetic tree was constructed using the neighbor-joining method [25].

Based on SNPs of the TRAP gene, the population structure of 110 *P. falciparum* isolates was inferred using the Bayesian clustering algorithm implemented in STRUCTURE software v. 2.3.3 [26]. STRUCTURE was run for *K* (a user-defined number of clusters) = 3 for 10,000 Monte Carlo Markov Chain (MCMC) iterations after a burn-in period of 10,000 using an admixture model and correlated allele frequencies.

Fst values [27] for three population pairs (Ratchaburi and Tak, Tak and Gambia, and Gambia and Ratchaburi) were calculated using the population genetics software package Genepop 4.2 [28]. The negative Fst values were regarded as 0 in this study.

## Supporting Information

Figure S1 **A sliding window analysis of Tajima’s** ***D*** **.** A sliding window analysis of Tajima’s *D* statistic across the entire TRAP gene for 32 Thai *P. falciparum* isolates was conducted using a sliding window size of 250 bp and a step size of 10 bp. The locations of four TRAP domains, signal sequence (SS), von Willebrand factor A domain (A domain), thrombospondin type 1 domain (TSP-1), and transmembrane domain (TM) were indicated by thick horizontal lines.
(TIF)
