# Supplementary figures and images for: Diversifying Selection on the Thrombospondin-Related Adhesive Protein (TRAP) Gene of Plasmodium falciparum in Thailand
Source: PLoS One. 2014 Feb 28;9(2):e90522. doi: 10.1371/journal.pone.0090522 (PMC3938765; doi:10.1371/journal.pone.0090522)

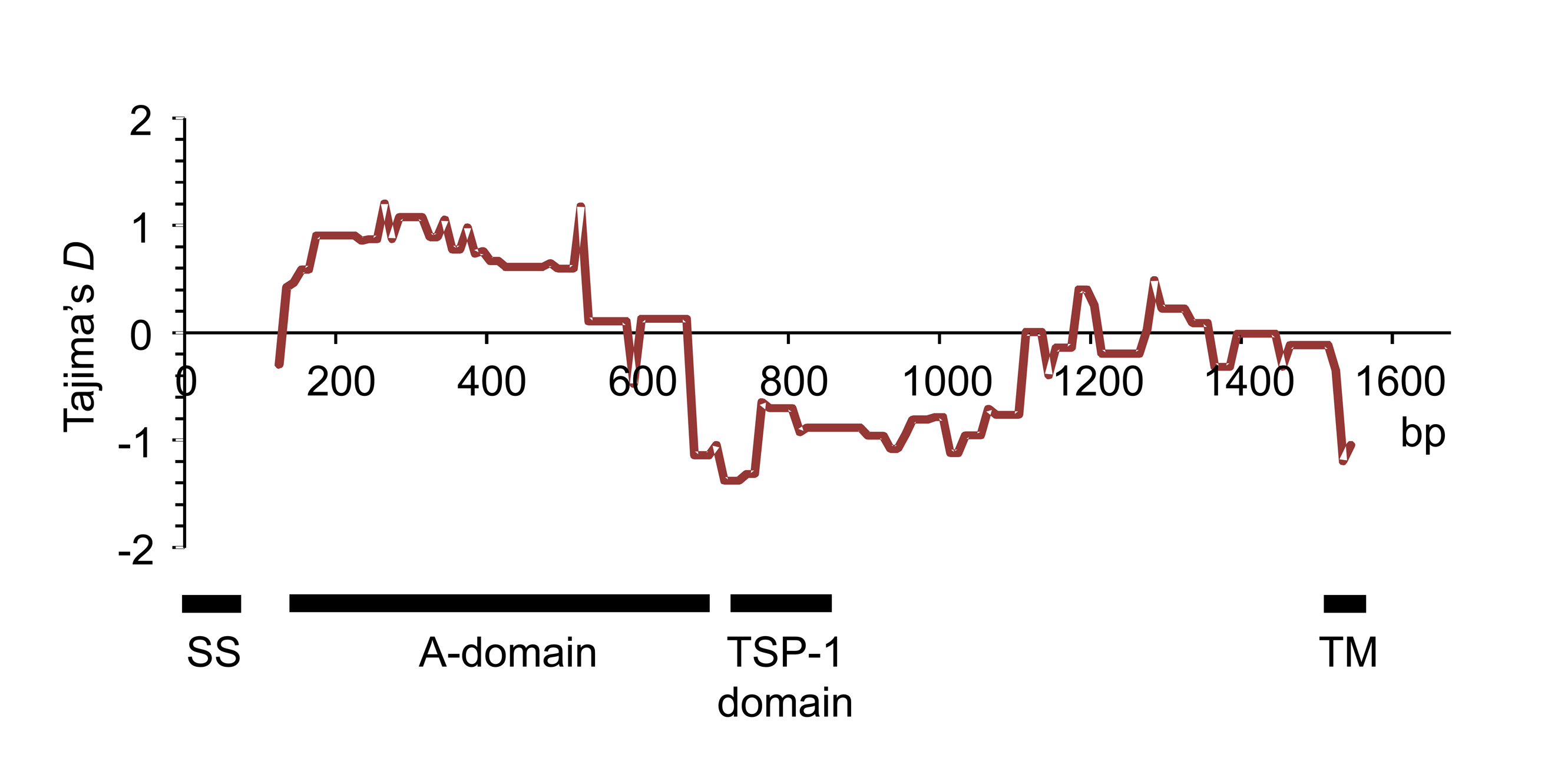

Supplement: Figure S1 — A sliding window analysis of Tajima’s D . A sliding window analysis of Tajima’s D statistic across the entire TRAP gene for 32 Thai P. falciparum isolates was conducted using a sliding window size of 250 bp and a step size of 10 bp. The locations of four TRAP domains, signal sequence (SS), von Willebrand factor A domain (A domain), thrombospondin type 1 domain (TSP-1), and transmembrane domain (TM) were indicated by thick horizontal lines. (TIF) [file pone.0090522.s001.tif]
